# A Novel Method to Predict Genomic Islands Based on Mean Shift Clustering Algorithm

**DOI:** 10.1371/journal.pone.0146352

**Published:** 2016-01-05

**Authors:** Daniel M. de Brito, Vinicius Maracaja-Coutinho, Savio T. de Farias, Leonardo V. Batista, Thaís G. do Rêgo

**Affiliations:** 1 Departamento de Informática, Centro de Informática, Universidade Federal da Paraíba, João Pessoa, Brazil; 2 Centro de Genómica y Bioinformática, Facultad de Ciencias, Universidad Mayor, Santiago, Chile; 3 Departamento de Biologia Molecular, Centro de Ciências Exatas e da Natureza, Universidade Federal da Paraíba, João Pessoa, Brazil; 4 Instituto Vandique, João Pessoa, Brazil; 5 Beagle Bioinformatics, Santiago, Chile; University of the West of England, UNITED KINGDOM

## Abstract

Genomic Islands (GIs) are regions of bacterial genomes that are acquired from other organisms by the phenomenon of horizontal transfer. These regions are often responsible for many important acquired adaptations of the bacteria, with great impact on their evolution and behavior. Nevertheless, these adaptations are usually associated with pathogenicity, antibiotic resistance, degradation and metabolism. Identification of such regions is of medical and industrial interest. For this reason, different approaches for genomic islands prediction have been proposed. However, none of them are capable of predicting precisely the complete repertory of GIs in a genome. The difficulties arise due to the changes in performance of different algorithms in the face of the variety of nucleotide distribution in different species. In this paper, we present a novel method to predict GIs that is built upon mean shift clustering algorithm. It does not require any information regarding the number of clusters, and the bandwidth parameter is automatically calculated based on a heuristic approach. The method was implemented in a new user-friendly tool named MSGIP—*Mean Shift Genomic Island Predictor*. Genomes of bacteria with GIs discussed in other papers were used to evaluate the proposed method. The application of this tool revealed the same GIs predicted by other methods and also different novel unpredicted islands. A detailed investigation of the different features related to typical GI elements inserted in these new regions confirmed its effectiveness. Stand-alone and user-friendly versions for this new methodology are available at http://msgip.integrativebioinformatics.me.

## Introduction

In the history of biological systems, many factors have played an important role in the evolutionary success of life. The possibility of generating diversity and spreading it across nature is one of the most important characteristics of life. In the origin of biological system, the capacity to exchange genetic novelties between the first forms of life was very important for survival and evolution of organisms or quasispecies [1]. During the ancient period, the horizontal gene transfer was the primary mode of spreading genetic diversity and this contributed strongly to the formation of the first cellular lineages [1]. However, together with the emergence of the first cells, it gave rise to cellular mechanisms to avoid the massive horizontal gene transfer (HGT) and preserve the genome integrity. From this period, vertical gene transfer has become the main process for transmission of genetic material into organisms. Nevertheless, the horizontal gene transfer maintains an important role in the evolution of modern organisms, promoting a rapid spread of important genetic novelties to specific conditions, contributing to the fast adaptation of many organisms. The identification of horizontal gene transfer events can aid in the comprehension of the evolutionary processes and their consequences in the ecological relationship between organisms, as well as in human and animal health.

Regions acquired by HGT are known as genomic islands (GI) and are associated with sequences that are acquired after the emergence of the vertical gene transfer as the main mechanism of genetic heritage [2]. These regions, depending on the time of transmission and the evolutionary distance between the donor and acceptor, can present significant differences in their nucleotides composition and the organization of their biological information, which makes the identification of horizontal gene transfer events in genomics analysis possible.

It is estimated that the bacteria *Escherichia coli* has acquired at least 17% of its genes through HGT [3]. This phenomenon is also observed in higher eukaryotes, with many examples of HGT from prokaryotes-to-eukaryotes and even between eukaryotic organisms [4–7]. These transferred regions are exchanged using transposons, phages and plasmids as classical vectors [8, 9]. In general, the acquired genes are responsible for the birth of important new functions and adaptations associated with pathogenicity (pathogenicity islands, PAIs), antibiotics resistance, symbiosis, degradation and metabolism. These new functions highlight the importance of HGTs in medicine, environment and industry [10]. The identification of these acquired genes is essential for the development of new vaccines, medicines and to understand environmental changes.

### Current methodologies for genomic islands detection

Genomic islands are genomic regions exchanged between different organisms. This transfer may result in a characteristic different G+C content and codon usage in that region in comparison to the rest of the genome [11]. The genes of a particular species are normally similar in their base composition and patterns of codon usage, in a way such that the sequence fragments acquired horizontally from other organisms can be distinguished computationally [12]. Current methods for the GI prediction based on sequence composition depends strongly on this fact. However, it leads to the non-identification of genomic islands in which the genomes of both donor and receptor organisms have similar or identical G+C content [3].

The identification of these genomic signatures can be represented using a metric. Two different commonly used measures are based on the G+C content and oligonucleotides composition (k-mers) [2]. In both cases, genome fragments (windows) are usually considered to measure the compositional bias and to compare it with the expected value for the entire genome, which corresponds to the whole sequence of composition average. Regions that differ from the rest of the genome are considered as GIs. Despite the windows being widely used, it is difficult to adjust their sizes, since small values lead to a large statistical fluctuation and higher values lead to a low resolution, making it impossible to detect small variations in the GC content [13]. Among the main methods that use this approach, we can highlight: SIGI-HMM [14], IslandPath [15], PAI-IDA [16], Centroid [17] and Alien_Hunter [18]. In 2004, Zhang and Zhang [13] proposed a method for the calculation of the G+C content of a genomic region without the use of windows, but a region of interest should be defined for the method be applied in GIs identification.

Other methods make use of multiple full genomic sequences of closely related phylogenetic species in order to investigate the presence of GIs. The full genome is aligned between two or more closely related organisms, and regions that are only present in the genome of interest are considered. This approach may be limited by the small number of sequences available for the group of organism under study. Also, it is important to note that the use of phylogenetically distant genomes can potentially result in the prediction of false positive GIs [2], while the use of closely related organisms can fail in the detection of recently transferred GIs that are acquired before the genomes start to differ from each other, leading to false negative islands. Among the main methods that use this approach we can highlight: MibolomeFinder [19] and IslandPick [20]. Finally, some tools have the option to use multiple methods for the prediction of genomic islands, allowing its visualization and comparison. Examples are EDIG [21], GIST [22] and IslandViewer [23].

Even with the availability of a broad range of methods, none of them are capable of predicting precisely the complete repertory of GIs in a genome. The difficulties arise due to the changes in performance of different algorithms in the face of the variety of nucleotide distribution in different species. It makes necessary the development of programs to combine various methods in order to increase the predictive accuracy in predicting GIs. This way, it is equally opportune to elaborate novel methods to employ different approaches for GI prediction in order to take full advantage of the benefits gained from identifying these regions. In this paper, we propose an alternative method for the prediction of GIs in bacteria using mean shift clustering algorithm. The mean shift does not require the definition of the number of clusters for the operation, but needs the bandwidth parameter to be set, which influences the number of clusters formed and affects the speed of algorithm convergence. The definition of bandwidth parameter is critical, strongly influencing the result of the algorithm. Thus, a heuristic was developed for automatic calculation. The method was implemented in a new tool named MSGIP—*Mean Shift Genomic Island Predictor*, freely available at http://msgip.integrativebioinformatics.me.

## Materials and Methods

### Clustering using mean shift

Mean shift is a non-parametric clustering algorithm that neither requires the prior definition of the number of clusters nor restricts the shape of the clusters [24]. The density estimation forms the basis of mean shift. For each data point, also referred to as feature vector, the algorithm executes a gradient ascend on local estimated density until its convergence. The stationary points represent density modes. The mathematical formulation is described as follows [25]:

Given a set of *n* data points **x**_*i*_ in *d*-dimensional space, where *i* = 1, …, *n*, the multivariate density estimation with kernel *K*(**x**), computed in the point **x** can be defined as follows:

$$\hat{f}\left(x\right)=\frac{1}{nh^{d}}\sum_{i=1}^{n}K\;\left(\frac{x-x_{i}}{h}\right)$$ (1)

where *h* is called the bandwidth and defines the kernel size.

The kernel function is defined as *K*(**x**) = *c*_*k*, *d*_ *k*(‖**x**‖^2^), where *c*_*k*, *d*_ is the normalization constant that integrates *K*(**x**) to 1, and *k*(*x*) is called kernel profile. Taking the gradient of the density estimator defined in Eq 1, we have the equation below after some algebraic manipulation, assuming the function, *g*(*x*) = −*k*′(*x*):

$$\nabla \hat{f}\left(x\right)=\underset{\text{first term}}{\underset{︸}{\frac{2c_{k,d}}{nh^{d+2}}\;\left[\sum_{i=1}^{n}g\;\left({\left\|\frac{x-x_{i}}{h}\right\|}^{2}\right)\right]}}\;\underset{\text{second term}}{\underset{︸}{\left[\frac{\sum_{i=1}^{n}x_{i}g\;\left({\left\|\frac{x-x_{i}}{h}\right\|}^{2}\right)}{\sum_{i=1}^{n}g\;\left({\left\|\frac{x-x_{i}}{h}\right\|}^{2}\right)}-x\right]}}$$ (2)

The first term of Eq 2 is proportional to Eq 1, and the second term is the mean shift vector **m**_*h*_(**x**), described below:

$$m_{h}\;\left(x\right)=\frac{\sum_{i=1}^{n}x_{i}g\;\left({\left\|\frac{x-x_{i}}{h}\right\|}^{2}\right)}{\sum_{i=1}^{n}g\;\left({\left\|\frac{x-x_{i}}{h}\right\|}^{2}\right)}-x$$ (3)

**Fig 1:**
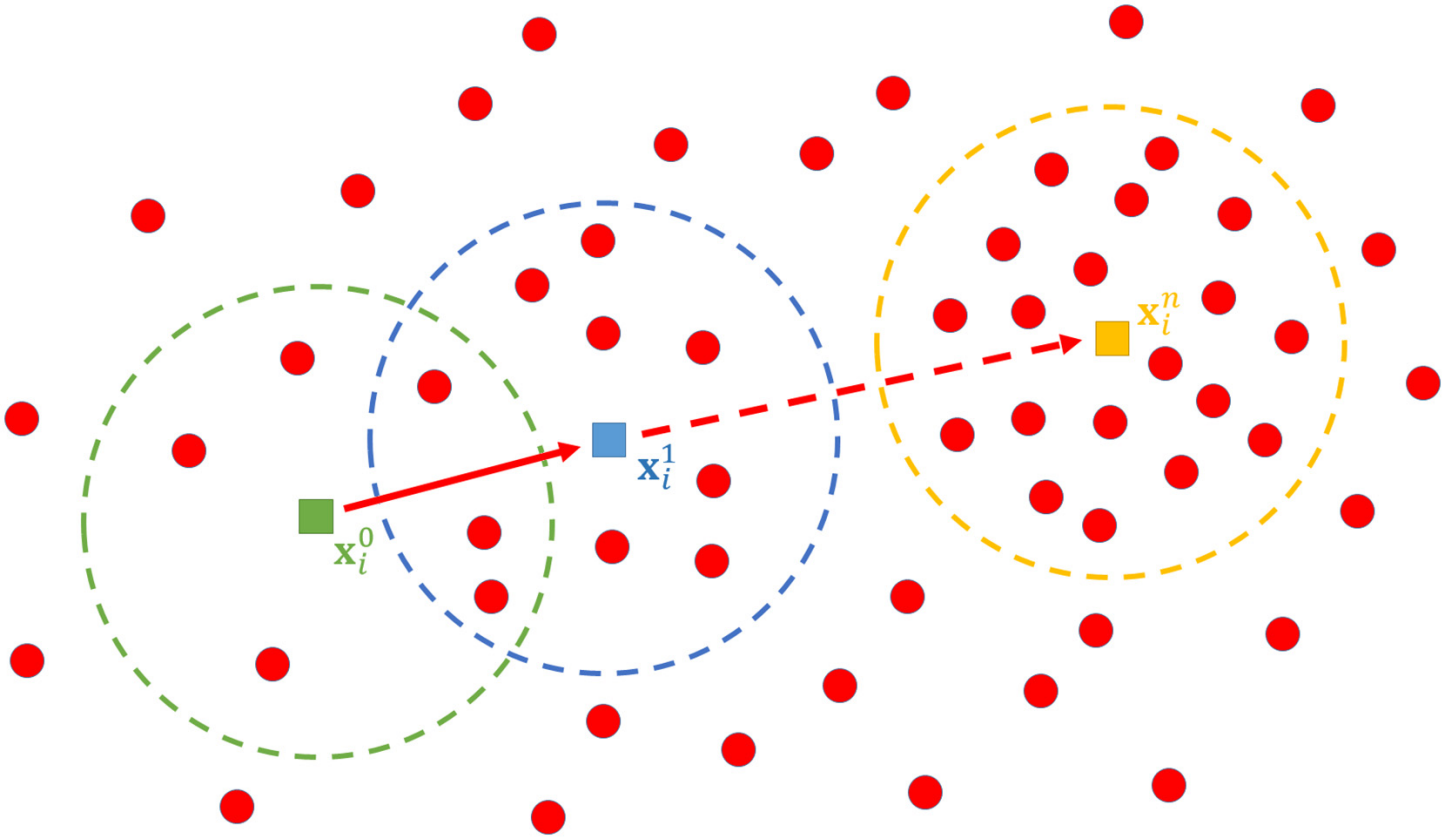
Mean shift algorithm procedure for a data point x_*i*_. The bold filled circles with arrows represent the iteration, while the pointed circles represent the window used in density estimation until the convergence is achieved at the *n*th iteration.

The mean shift vector always points in the direction of the maximum increase of density. The algorithm procedure performed for a given data point **x**_*i*_ is described in the following points:

1. Calculates the mean shift vector $m_{h}\left(x_{i}^{t}\right)$;
2. Move the density estimation window $x_{i}^{t+i}=x_{i}^{t}+m_{h}\left(x_{i}^{t}\right)$;
3. Repeat the above steps until convergence, i.e. when, $x_{i}^{t+1}-x_{i}^{t}<\epsilon$.

where the superscript *t* is the current procedure iteration, and *ϵ*, the threshold. The procedure application for a data point **x**_*i*_ is illustrated in Fig 1

The number of clusters is automatically generated through the mode calculation of each point. Further, points that have the same mode are assigned to the same cluster.

### Automatically setting the optimal bandwidth size

A common problem found in most partitional clustering algorithms is the necessity to specify a priori the number of clusters to be formed. However, mean shift does not require this parameter; it only requires the bandwidth length to be defined beforehand. The definition of this parameter is not a trivial task. Here, we choose to develop a heuristic to automatically calculate this parameter through the insertion of artificial genes. First, the user defines a fixed number of artificial fragments that will be extracted from the genome of different bacteria and inserted into the genome of interest. The fragments’ sizes might be similar to the length defined by the user for the window size parameter. Then, the mean shift algorithm is executed until all windows from the original genome are grouped into different clusters. The bandwidth value is decreased and the algorithm is executed again. The process is repeated until the algorithm sets all the artificially inserted windows into different clusters. It is believed at this moment that the windows that host genes potentially acquired horizontally will also be kept in separate groups from the rest of the genomes, allowing them to be identified.

The selection process of artificial windows is described as follows. For each one of the nucleotides (A, T, C, G), the mean and standard deviation of its sum are calculated in all windows of the investigated genome. The user might specify the length of window *j*. In a set of genomes, artificial windows are selected in accordance with the following criteria:

$$\sum_{i=0}^{j}Y_{i}<\bar{X}-c\sigma_{x}\;\text{or}\;\sum_{i=0}^{j}Y_{i}>\bar{X}+c\sigma_{x}$$ (4)

where *c* is a positive constant bigger or equal to 1, that might be provided as input to this method.

The higher the selected coefficient for standard deviation, the more distant is the artificially inserted window in comparison to the original genome. One should also ensure that there is no similarity between the artificially inserted windows. In this work, we consider artificial sequences that differ by more than 7.5%, in the sum of their four nucleotides, from the other selected artificial sequences.

### Identification of genomic islands

It is expected that the windows of genes acquired horizontally differ in their nucleotides composition in relation to the rest of the host genome sequence. After the mean shift clustering execution using the ideal bandwidth value, these windows will be grouped separately in relation to the rest of the genome.

According to Hacker and Kaper [3], the sizes of genomic islands typically vary between 10kb and 200kb. However, here we consider clusters containing up to 200kb, varying according to the size of the selected windows. We choose this approach due to the variation in the sizes of genomic islands, so that a genomic island can correspond to the length of multiple consecutive windows. Roos and Van Passel [26] pointed that a unique bacterium can be responsible for multiple horizontal transferences in a unique receptor organism. Because of that, we considered GIs as those clusters in which the sum of windows does not surpass 200kb, regardless of the order in which they appear. Other values were also tested and nearby numbers did not influence the results, while small or large values were influenced negatively.

### Datasets

In this work, we have used the genome sequence from bacteria with known genomic islands. The full sequences for each species or strain were downloaded from the Bacteria database from NCBI (ftp.ncbi.nih.gov/genomes/Bacteria) and are listed in Table 1.

**Table 1:** Genomes used for testing mean shift and other related algorithms.

Genome	Length(Mb)	Accession Number	References
*Corynebacterium glutamicum* ATCC 13032	3.309	NC_003450.3	[27]
*Vibrio vulnificus* CMCP6 chromosome I	3.281	NC_004459.3	[28]
*Rhodopseudomonas palustris* CGA009	5.459	NC_005296.1	[29]
*Streptococcus mutans* UA159	2.032	NC_004350.2	[30]
*Vibrio cholerae* O1 biovar El Tor str. N16961 chromosome II	1.072	NC_002506.1	[31]
*Vibrio vulnificus* YJ016 chromosome I	3.354	NC_005139.1	[32]
*Mycoplasma genitalium* G37	0.580	NC_000908.2	[33]
*Rickettsia prowazekii* str. Breinl	1.109	NC_020993.1	[34]

## Results and Discussion

In this section, the mean shift method is applied in bacterial genomes, where genomic islands were previously described in the literature. The G+C cumulative profile [13] is used to visualize the obtained results. The curve generated by this approach can represent different interesting characteristics of a genome sequence. Zhang and Zhang [13] showed that the G+C content inside genomic islands is reasonably homogeneous in comparison to the rest of the host genome, in a way that these regions are represented in the curve as almost straight lines. A jump or a fall in the curve indicates an abrupt decrease or increase in the G+C content, pointing to a potential source of horizontal transfers in that region.

We applied variations on the set of parameters for this method to find the best option. Finally, we have obtained consistent results with all tested genomes when the following parameters were used: (i) window length: 50kb; (ii) quantity of artificially inserted genomic sequences fixed in 5; and (iii) a standard deviation of 1.

**Fig 2:**
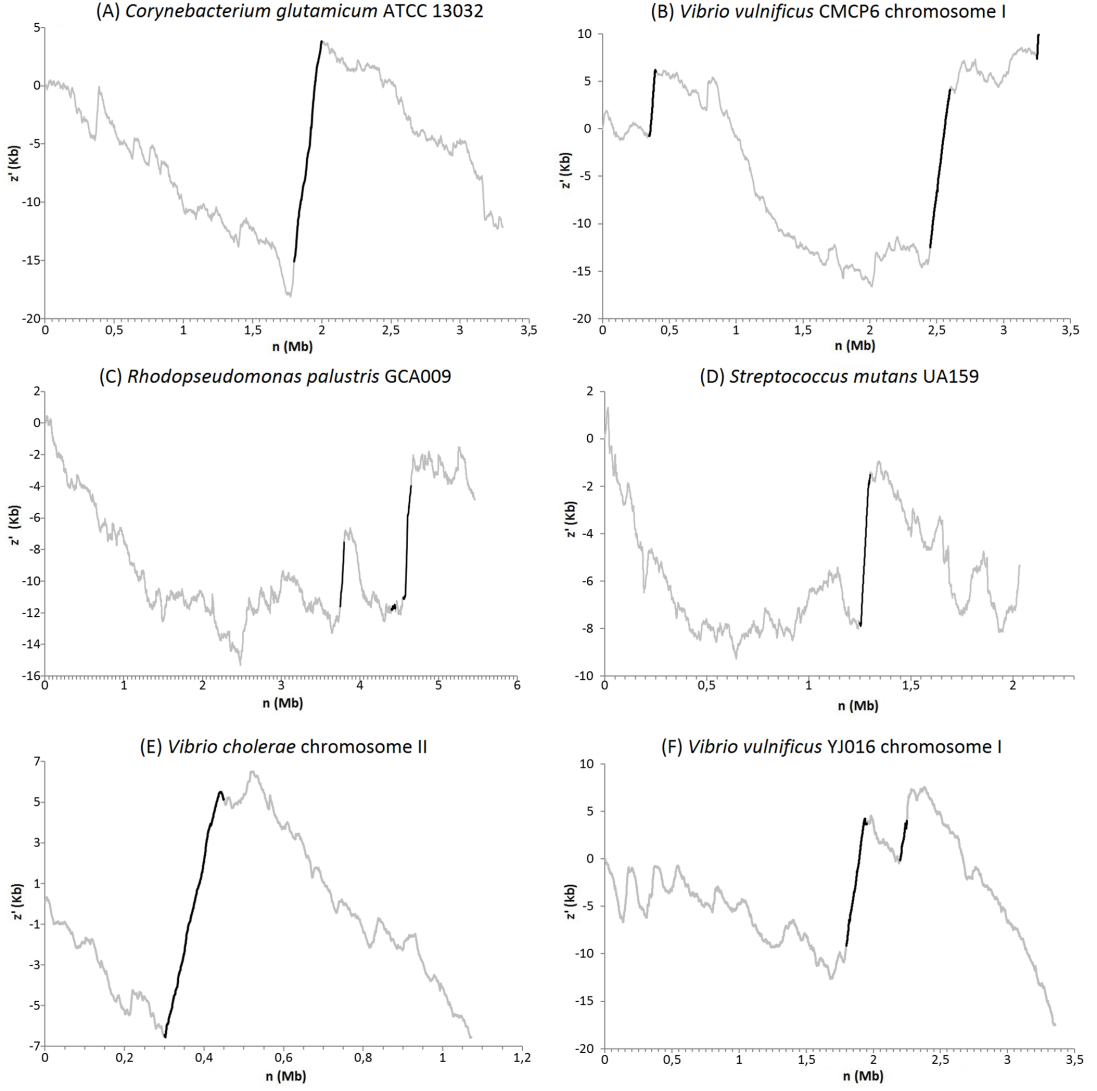
Graphics representing the *z*′ curves for following six different bacteria genomes. (A) *Corynebacterium glutamicum* ATCC 13032, (B) *Vibrio vulnificus* CMCP6 chromosome I, (C) *Rhodopseudomonas palustris* CGA009, (D) *Streptococcus mutans* UA159, (E) *Vibrio cholerae* chromosome II e (F) *Vibrio vulnificus* YJ016 chromosome I. Black lines represents the genomic islands identified by the mean shift method.

In Table 2, we have listed all genomic islands detected by the mean shift and other methods for all genomes and species listed in Table 1. The *z*′ curve for every studied genome can be observed in Fig 2. The regions containing a genomic island are highlighted by black lines in the graphic.

**Table 2:** Genomic islands detected by mean shift and its comparison with other methods previously used for each species.

Genome	Identifier	Detected GI (Mb) (This work)	Corresponding GI (Mb) (Other Methods)	Characteristics
***Corynebacterium glutamicum*** **ATCC 13032**	CGGI01	1.800–2.000	1.776–1.987 [13]	Hypothetical proteins (with unknown function)
***Vibrio vulnificus*** **CMCP6 chromosome** I	VVCGI01	0.350–0.400	0.355–0.395 [13]	Hypothetical proteins and invasion-associated proteins
	VVCGI02	2.450–2.600	2.438–2.605 [13]	Invasion-associated proteins
	VVCGI03	3.250–3.281	3.248–3.281 [13]	Transporter protein, transposase, phage and hypothetical proteins
***Rhodopseudomonas palustris*** **CGA009**	RPGI01	—	2.481–2.564 [35]	IV secretion genes for conjugal transfer of DNA, arsenate reductase pump modifier and an arsenical pump membrane protein
	RPGI02	3.750–3.800	3.729–3.807 [35]	Hypothetical proteins
	RPGI03	4.400–4.450	—	Hypothetical proteins and flagellar proteins
	RPGI04	4.550–4.650	4.578–4.678 [35]	Multidrug efflux and transporter related genes
***Streptococcus mutans*** **UA159**	SMGI01	1.250–1.300	1.250–1.300 [36, 37]	TnSMU2 (nonribosomal peptide synthetases (NRPS), polyketide synthases (PKS), accessory proteins, transporters, and transcription regulators)
***Vibrio cholerae*** **chromosome II**	VCGI01	0.300–0.450	0.302–0.436 [38]	Chloramphenicol acetyltransferase, killer protein, antidote protein, haemagglutinin, others copies of acetyltransferase and Hypothetical protein
***Vibrio vulnificus*** **YJ016 chromosome I**	VVYGI01	—	0.159–0.167 [38]	Lactoglutathione lyase
	VVYGI02	1.800–1.950	1.757–1.936 [38]	Hypothetical proteins and transposases
	VVYGI03	2.200–2.250	—	Hypothetical proteins, region started and finished

### *Corynebacterium glutamicum* ATCC 13032

*Corynebacterium glutamicum* ATCC 13032 is a gram-positive bacterium that is important for industrial production of amino acids [39], with a genome size estimated in 3.309 Mb. Here, the mean shift method identified one genomic island (Table 2). The region of 200kb corresponding to the position 1.800–2.000 Mb is represented in Table 2. The *z*′ curve generated for this genome is displayed in Fig 2A. This genomic island was predicted in the same region as described by [13], where 82.9% of the identified genes encode hypothetical proteins (with unknown function), while for the remainder of the genome, only 26.6% encodes these proteins. This difference indicates that the genes present in these regions were acquired from other organisms, and can therefore be described as GIs.

### *Vibrio vulnificus* CMCP6 chromosome I

*Vibrio vulnificus* CMCP6 is a pathogenic estuarine bacterium with a genome estimated in 3.281 Mb, which affects a human being’s underlying hepatic diseases and other immune-compromised conditions [40]. As listed in Table 2, the mean shift method identified three different genomic islands in this genome. All of them correspond to previously described genomic islands by [13]. The GI VVC01 corresponds to the position 0.350–0.400 Mb (see Table 2 and Fig 2B) and host genes associated with hypothetical proteins and invasion-associated proteins; and the GI VVC02 corresponds to the region 2.438–3.605 Mb and also the host genes related to invasion-associated proteins. Finally, a last island was identified at the end of the genome (position 3.250–3.281 Mb) and host site-specific recombinase, phage, integrase, transposase, multidrug transporter and hypothetical proteins.

### *Rhodopseudomonas palustris* CGA009 CMCP6 chromosome I

*Rhodopseudomonas palustris* CGA009 is a gram-negative bacterium, recognized by its versatile metabolism to produce energy by using light and inorganic and organic compounds [29]. For this bacterium, the mean shift method identified three different genomic islands (see Table 2). Two of them were previously identified by [35], while the remaining were identified for the first time. The *z*′ curve for this genome is displayed in Fig 2C. The island RPGI01, corresponds to several hypothetical proteins, typical to a genomic island as described by [2]. However, this genomic island was not identified by our method.

The region RPGI02 hosts genes associated with hypothetical proteins and consist of a previously described genomic island [35]. RPGI03 was also predicted by [35], and hosts a high presence of hypothetical proteins, as well as transport proteins associated with the flagellar system. The last predicted genomic island hosts proteins associated with two multidrug efflux and transport related genes, suggesting that horizontal gene transfer may play a role in the antibiotic resistance of *R. palustris*.

### *Streptococcus mutans* UA159

*Streptococcus mutans* UA159 is a gram-positive bacterium, and it is known to be the leading cause of dental caries [30]. The mean shift method identified only one genomic island in this bacterium (Table 2 and Fig 2D). The GI consists of the region *TnSmu2*, which is known to have much lower G+C content than the rest of the genome [36, 37]. This region carries genes coding for non-ribosomal peptide synthetases, polyketide synthases, and accessory proteins that are responsible for the biosynthesis of the pigment mutanobactin carried by *S. mutans*. Mutational analysis further demonstrated that this gene cluster (named the *mub* locus) is involved in oxygen tolerance, H2O2 resistance, and biofilm formation in *S. mutans*[41].

### *Vibrio cholerae* chromosome II

*Vibrio cholerae* is the etiological agent of the cholera disease [42]. For this bacterium, the mean shift identified only one region as a potential genomic island (Table 2 and Fig 2E). This island’s host genes are associated with chloramphenicol acetyltransferase, killer protein, antidote protein, haemagglutinin and other copies of acetyltransferase. Also, it was observed many hypothetical and conserved hypothetical proteins. This island was also previously predicted by [38].

### *Vibrio vulnificus* YJ016 chromosome I

As previously described, *Vibrio vulnificus* is a human pathogenic bacterium. For this different strain, we identified two different genomic islands with the mean shift method (Table 2 Fig 2F). The GI VVY02 was previously described by [38]. The other region identified only by our method (VVY03) hosts several hypothetical proteins and phages, and begins and ends with transposases, which are known to be associated with genomic islands insertions. The mean shift method was not capable of identifying GI VVY01 (position: 0.159–0.167 Mb), as described in [38].

### *Mycoplasma genitalium* G37 e *Rickettsia prowazekii*

It has been reported that some bacterium does not have genomic islands in its genome composition. This is the case with *Mycoplasma genitalium*[13], the smallest known genome of any free-living organism, which was originally isolated from urethral specimens of patients with non-gonococcal urethritis. It lives in a parasitic association with ciliated epithelial cells of primate genital and respiratory tracts [33]. *Rickettsia prowazekii*, the agent of epidemic typhus, and a potential biothreat agent [43], also does not have any genomic islands [44]. We used the mean shift method on the genome sequence of both bacteria to evaluate the method efficiency and the potential prediction of false positive islands. As expected, genomic islands were not detected for both bacteria.

### MSGIP—*Mean Shift Genomic Island Predictor tool*

The mean shift method described here was implemented in a standalone and user-friendly tool named MSGIP—*Mean Shift Genomic Island Predictor*. MSGIP was developed in Java and is compatible with any operating system with Java Runtime Environment installed. MSGIP outputs the predicted GIs in a text-like format that can be further saved into a file. The usage requires only a FASTA “.fna” file, containing the completely genomic sequence of the investigated bacteria. The user has to set the three parameters discussed in the text or, alternatively, he can use the default parameters tested by the authors in this study. The dataset of bacteria used to extract the artificial fragments is provided by MSGIP, but the user can add other sequences (in.fna format) to the folder “genomes” containing the sequences file. MSGIP source code and friendly version are freely available and can be downloaded at: http://msgip.integrativebioinformatics.me.

## Conclusions

This work presents a new method for genomic island prediction based on the mean shift clustering algorithm. Our results prove that this new method can produce results that are consistent with other methods described in the previous literature. We consider the simplicity and easy implementation of this method as an advantage in comparison to other methods used for genomic island prediction, since the current model uses only the base vector (A, T, C, G) of its own genome sequence as a measure of composition. However, the calculation of bandwidth parameter must be studied in greater detail in order to identify the possible bias generated by artificially selected genes during the determination of bandwidth parameter. We expect this method to be used in discovering new GIs, which are not predicted by other methods and can also be used in combination with other methods.
